# Increased activity of the complement system in cerebrospinal fluid of the patients with Non-HIV Cryptococcal meningitis

**DOI:** 10.1186/s12879-016-2107-9

**Published:** 2017-01-04

**Authors:** Lei Shen, Jianming Zheng, Yan Wang, Mengqi Zhu, Haoxiang Zhu, Qi Cheng, Qian Li

**Affiliations:** 1 Department of Thoracic Intensive Care Units, Shanghai Pulmonary Hospital, Shanghai, China; 2 Department of Infectious Diseases, Huashan Hospital, Fudan University, Shanghai, China; 3 Central Laboratory, Huashan Hospital, Fudan University, Shanghai, China

**Keywords:** Cryptococcal meningitis, Complement, Cryptococcus

## Abstract

**Backgrounds:**

Cryptococcal meningitis (CM) has been known to lead to significant morbidity and mortality. The relative contribution of the complement system in protection and pathogenesis during CM remains largely unknown. The purpose of this study was to evaluate the baseline complement component profiles in human cerebrospinal fluid (CSF) and plasma from non-HIV patients with CM, and therefore to provide insights of possible roles of the complement system in CM.

**Methods:**

CSF and blood samples from forty two CM patients not infected with HIV and thirteen non-CM control patients (Ctrl) were retrospectively selected and evaluated from the patients admitted to the hospital with a suspected diagnosis of CM. CSF and blood samples were collected at the admission. Enzyme-linked immunosorbent assay (ELISA) for complement components, cytokine IL-12 and western blot for C3 activation were performed on CSF and plasma samples. The levels of complement C1q, factor B (FB), mannose binding lectin (MBL), C2, C3, C4, C5, C4 binding protein (C4BP), Factor I (FI), Factor H (FH), sC5b-9 in CSF and plasma samples were compared. Pearson’s correlation coefficients were calculated on variables between complement components and the levels of total protein in the CSF samples.

**Results:**

Our data demonstrated that the CSF levels of complement components of C1q, FB, MBL as well as complement pathway factors sC5b-9 and complement regulator FH were all elevated in patients with CM as compared to the controls, CSF C3 breakdown products iC3b were found in both CSF and plasma samples of the CM patients. A positive correlation was found between the levels of CSF protein and MBL, C1q or FB.

**Conclusions:**

The activity of the complement system in CSF was increased in non-HIV patients with CM. C1q, MBL and FB are the important participants in the complement activation in CM. The relative contribution of each of the specific complement pathways and complement cascades in protection and inflammation resolution against CM warrant further investigation.

## Background

Cryptococcal meningitis (CM) is one of the most common diseases in HIV patients and other immune-compromised patients [1]. Over 1 million CM cases with 600,000 deaths per year in HIV patients worldwide have been reported [2]. Additionally, non-HIV related CM also contributes a significant mortality and morbidity in the developed world despite advances in antifungal therapy [1]. In China, the main pathogen of cryptococcal infections is *C. neoformans*, resulting in pulmonary cryptococcosis that has a predilection to disseminate to the central nervous system (CNS) leading to life threatening meningoencephalitis [3].

The complement system is well known as a major component of the host innate immune defense system against infection. Activation of the complement system in response to invading pathogens is initiated through the classical (CP), alternative (AP) and lectin (LP) pathways. Activation results in C3 cleavage, releasing anaphylatoxins C3a and C5a, and formation of a membrane attack complex to lyse the target cells. Cleavage of C3 generates the key opsonins, C3b and iC3b. These proteins tag the pathogens for phagocytosis [4]. The complement system has the inhibitory proteins to regulate the location and efficacy of complement activation. Key fluid phase complement regulators include Factor H (FH), Factor I (FI), C4-binding protein (C4BP) and C1 inhibitor (C1INH).

It has been suggested that the complement system is of central importance in host defense against bacterial meningitis. Mice deficient in C1q and C3 demonstrated a decreased survival and enhanced growth of pneumococci in cerebrospinal fluid (CSF) after induction of meningitis [5]. Complement factors C1q, MBL, C3a, iC3b, C5a, sC5b-9 and FH were all elevated in CSF of patients with bacterial meningitis as compared to the controls [6]. Depletion of C3 or elevated C5a levels in CSF of patients with pneumococcal meningitis was associated with poor clinical prognosis [7, 8].

In contrast to bacterial meningitis, most fungal microorganisms cause a chronic instead of an acute or subacute meningitis [9]. Investigations from animal model and human patients have shown the importance of the complement system against cryptococcal infections [10]. Mice lacking hemolytic complement activity in their sera were sensitive to *C.neoformans* [11]. Guinea pigs and mice treated with cobra venom (depletion of C3 to C9) showed reduced ability to clear *C.neoformans* from extraneural sites [12]. *C5* ^*−/−*^ mice were more susceptible to intravenously injected *C.neoformans*, and more easily developed acute, fatal cryptococcal pneumonia [13]. Patients with cryptococcal fungemia showed reduced levels of C3 and FB [14]. Brain sections from patients with CM did not show C3 binding to the pathogen [15]. Moreover, both the classical and alternative complement pathways play a critical role in opsonophagocytosis of *C.neoformans* by neutrophils [16]. In vitro experiments demonstrated that C5a-C5aR signaling guided neutrophils to migrate to *C.neoformans,* resulting in optimal phagocytosis and subsequent killing of the organisms [17]. The complement opsonization is required in the anti-cryptococcal activity of human dendritic cells and macrophages [18, 19]. These data suggest that the major functions of the complement system are to stimulate the chemotaxis of the phagocytic effector cells and enhance the uptake of cryptococcal cells by these phagocytes. Complement functionality can be crucial to fight off and kill invading *Cryptococcus.*

To our knowledge, no study has been reported which examines the complement system activity in CSF and plasma from CM patients. The purpose of this study was to investigate whether the complement system is activated systemically and/or intrathecally during CM. The levels of the complement components in CSF and plasma samples from CM patients were determined and compared with that in the patient control group who were diagnosed with pulmonary cryptococcosis without CNS infection and inflammation involvement.

## Methods

### Patient selection, clinical sample collection and processing

This study was carried out from November 2014 to December 2015 at Huashan Hospital, Shanghai China. Both lumbar CSF and plasma samples were simultaneously obtained from patients with a suspected CM diagnosis at admission for routine chemistry, cytology and culture analysis. Venous blood was collected from individuals for plasma and routine blood cell and culture analysis. The remaining CSF samples were centrifuged and single use aliquots of these samples were stored at −80 °C.

CM group and patient control group were retrospectively selected from the collected samples. CM group consisted of 42 patients who were ≥18 year old and not infected with HIV with a first episode of CM as diagnosed according to the criteria reported previously [20]. Confirmed cases were patients meeting the laboratory criteria with positive culture of *C. neoformans* or positive India ink smear of CSF. Possible cases were patient with positive cryptococcal antigen titer in undiluted CSF and met at least one of the following criteria : (1) abnormal laboratory tests or an increased open pressure (≥200 mmH_2_O) of CSF, (2) abnormalities of cranial imaging (Computerized Tomography or Magnetic Resonance Imaging) which could not be explained by other factors. Cryptococcal antigen in CSF was determined with the Latex-*Cryptococcus* antigen detection system (Immuno-Mycologics) [21].

Control CSF or blood samples were obtained from patients who presented to the hospital with suspicion of CM but subsequently tested negative for CM or any other pathogen in CSF with normal CSF leukocyte count and protein concentration. The control patients were diagnosed as pulmonary cryptococcosis based on histologic presence of the organism in lung biopsy specimens, or positive result of the serum cryptococcal antigen test with radiographic characteristics of pulmonary disease. [22]. The ethical and scientific committee of the College of Medicine, Fudan University approved this study.

### ELISA

Human Complement C1q, FB, MBL, C2, C3, C4, C5, C4BP, FH, FI, sC5b-9, and cytokine Interleukin (IL) -12 were measured with commercial ELISA kits (Cusabio Biotech, Wuhan, P.R. China, R&D Systems, Minneapolis, USA, Abcam, Cambridge, UK, QuidelSan Diego, USA). Experiment protocols were followed according to the manufacturer’s instructions.

### Western blot

For comparison of complement C3 activation in CSF and plasma samples, 20 μl of CSF and 2 μl of plasma samples were loaded on an SDS-PAGE gel. Samples were transferred to polyvinylidene difluoride (PVDF) membranes. The membranes were blocked and then incubated with mouse anti-human C3/C3b/iC3b/C3dg monoclonal antibody (1:1,000, Cedarlane, Ontario, Canada) in TBST (Tris-buffered saline supplemented with Tween 20) containing 5% dry milk at 4 °C overnight. Blots were then washed and incubated with goat anti-mouse IgG (1:15,000, LI-COR Bioscience, Nebraska, USA) diluted in TBST containing 2% dry milk. The membranes were developed with an Odyssey system (Li-COR Bioscience) according to the manufacturer’s protocol.

### Statistical analysis

Results were graphed and analyzed using Prism 5.0 (GraphPad). Comparisons between the CM and control groups were made using unpaired *t* tests or Mann–Whitney tests (when the variances were significantly different). Nominal data were analyzed with Fisher exact test or Chi *χ* ^2^ test. Pearson’s correlation coefficients were calculated on the variability between the levels of CSF complement components and CSF total protein. In all cases, a *p* value of <0.05 was set as the measure of significance.

## Results

### The clinical characteristics of participants

The study groups consisted of 42 CM patients and 13 patients diagnosed with pulmonary cryptococcosis without CNS infection and inflammation served as Ctrl group. As shown in Table 1, there were no significant differences in gender, age, blood routine test, CSF concentration of glucose and chloride between the two groups. However, WBC counts and concentrations of protein in CSF were significantly higher in CM group than that in the Ctrl group.

**Table 1:** The clinical characteristics of participants

	Ctrl	CM	*p* value
Gender, M/F	9/4	28/14	0.5740
Age (means ± SEM, years old)	46.38 ± 11.15	44.86 ± 14.14	0.7233
Blood WBC (means ± SEM, 10^6^/mm^3^)	6.98 ± 2.67	8.20 ± 3.75	0.2981
CSF WBC (median, 10^3^/mm^3^)	2	9	0.0022
CSF Protein (median, mg/l)	387.5	883	0.0006
Blood Sugar (median, mmol/l)	5.4	6.9	0.5631
CSF Glucose (median, mmol/l)	2.65	2.70	0.4762
CSF Chloride (median, mmol/l)	121	120	0.3703

CM: cryptococcal meningitis group

Ctrl: control group

### Complement components in CSF and plasma samples

#### The CSF concentrations of C1q, MBL and FB of CSF in CM were greatly elevated compared to that in Ctrl, and correlated with CSF protein concentrations

**Fig. 1:**
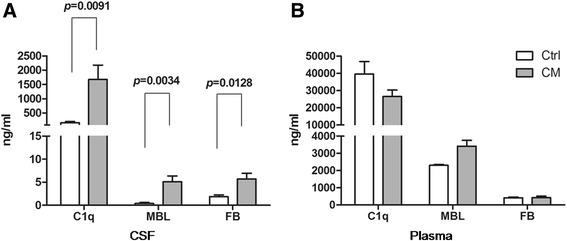
The concentrations of C1q, MBL and FB in plasma and CSF samples. The white bar represented Ctrl group and the gray bar represented CM group. **a** CSF concentrations of C1q, MBL and FB were significantly higher in the CM group than those in the Ctrl group. **b** There were no significant differences in plasma concentrations of three complement components among the Ctrl and CM groups

We first examined the concentrations of the complement classical pathway component (C1q), the lectin pathway (MBL) and the alternative pathway (FB) in plasma and CSF samples from CM and Ctrl group. The concentrations of C1q, MBL and FB in CSF samples were significantly higher in CM group than that in Ctrl group (Fig. 1a). The mean CSF concentration of C1q was 1673.14 ng/ml in the CM group, but only 162.72 ng/ml in the Ctrl group. The mean concentration of MBL was 5.11 ng/ml in the CM group, but 0.38 ng/ml in the Ctrl group. The mean concentration of FB was 5.73 ng/ml in the CM group, but 1.88 ng/ml in the Ctrl group. Moreover, CSF concentrations of C1q, MBL and FB were found to be correlated to CSF protein levels (r = 0.5804 for C1q, *p*-value =0.0479, r = 0.7061 for MBL, *p*-value = 0.0003, and r = 0.6716, *p*-value =0.009). However, There were no statistical significances of the plasma concentrations of C1q, MBL and FB between the CM and Ctrl groups (Fig. 1b), suggesting that increased concentrations of C1q, FB and MBL in CSF is a localized phenomenon.

#### The concentrations of complement components C2, C3, C4 and C5 in plasma and CSF samples

**Fig. 2:**
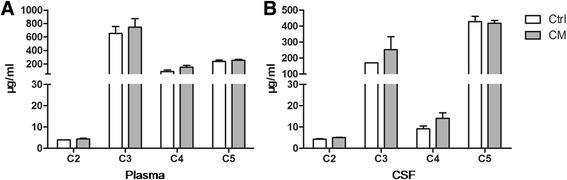
The concentrations of C2, C3, C4 and C5 were detected in both plasma (**a**) and CSF (**b**) samples. There were no significant differences between the CM and Ctrl groups

We further examined the concentrations of C2, C3, C4 and C5 in the plasma and CSF samples. There were no statistical significances of the concentrations of these complement factors in both plasma and CSF samples between the two groups (Fig. 2a and b).

#### C3 activation in CSF and plasma in CM

**Fig. 3:**
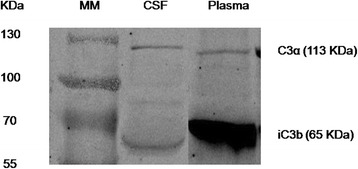
C3 cleavage fragments were analyzed by western blot. Approximately 20 μl of CSF and 2 μl of plasma samples were subjected to SDS-PAG. The immunoreactive bands corresponding to C3α and iC3b are denoted. Prominent iC3b band was found in the CSF and plasma samples in CM patients. A representative is shown

The complement system activation is mediated through the CP, AP and LP pathways. Each pathway converges at the C3 convertase level resulting in C3 cleavage. To further assess whether the C3 activation is involved in CM, levels of C3 breakdown products in CSF and those in simultaneously collected blood plasma samples were evaluated by Western blot. C3 cleavage fragments iC3b was detected in CM group from both plasma and CSF samples (Fig. 3).

#### The concentrations of sC5b-9 in plasma and CSF samples

**Fig. 4:**
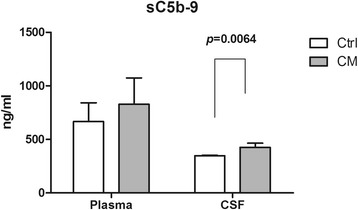
The concentrations of sC5b-9 in plasma and CSF. The concentration of sC5b-9 in CSF increased significantly in CM group compared to the Ctrl group and the *p* value was 0.0064

To determine the complement terminal pathway activation, we examined the concentrations of sC5b-9 in plasma and CSF samples in Ctrl and CM groups. There was no significant difference in plasma concentrations of sC5b-9 between the two groups. However, the CSF concentrations of sC5b-9 in CM group was significantly higher than that in the Ctrl group (*p* = 0.0064) (Fig. 4).

#### The concentrations of C4BP, FI and FH in plasma and CSF

**Fig. 5:**
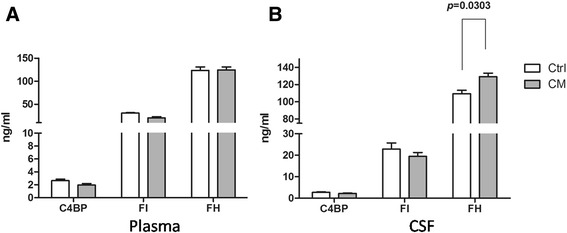
The concentrations of C4BP, FI and FH in CSF and plasma. **a** There were no significant differences in plasma concentrations of C4BP, FI and FH between the two groups. **b** There were no significant differences in CSF concentrations of C4BP and FI between the two groups. However, CSF levels of FH in CSF was significantly higher in CM group than that in Ctrl group

The complement system is regulated by some key fluid regulators, such as C4BP, FI and FH. We next measured the concentrations of these factors in plasma and CSF samples. There were no significant differences in the plasma concentrations of C4BP, FI and FH expressed between Ctrl and CM groups (Fig. 5a). The concentrations of C4BP and FI in CSF were also similar in both Ctrl and CM groups (Fig. 5b). However, the CSF concentrations of FH increased significantly in CM group compared to the Ctrl group (*p* = 0.0303) (Fig. 5b).

#### The levels of IL-12 in the plasma and in CSF samples

**Fig. 6:**
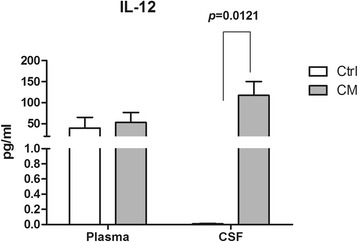
The levels of IL-12 in plasma and CSF samples. The plasma levels of IL-12 were similar between the Ctrl and CM groups, but the CSF levels of IL-12 significantly increased compared to in CM group that in the Ctrl group

We next investigated the levels of IL-12 because it may plays an important role in host defense in CM [23]. As shown in Fig. 6, though the plasma levels of IL-12 were similar between the Ctrl and CM groups, the CSF levels of IL-12 were significantly higher in CM group than that in Ctrl group (*p* = 0.0121) (Fig. 6).

## Discussion

In this study, we showed elevated CSF concentrations of C1q, MBL, FB, sC5b-9 and FH in CM patients compared to that in the control group and the common complement pathway factors iC3b in both CSF and plasma samples of CM patients. Our data suggest that the increased CSF complement system activity might indicate its important roles in host defense and pathogenesis in CM.

Though the importance of the complement system to *C. neoformans* infection is well established, it is not clear how the complement system responds to pathogens in CM clinically. The brain may represent its own characteristics not like other organ when infected with *Cryptococcus*. There are few publications in this field. Our data demonstrated that complement components including the initiators of three complement pathways (C1q, FB and MBL), other complement factors (C2, C3, C4, C5) and regulators (FH, FI, C4BP) were present in CSF in CM. Moreover, CSF levels of C1q, MBL, and FB were significantly higher compared with that in control group. In this study, we showed C3 and the complement terminal pathways were activated in CSF during CM. Cleavage of C3 generates the key opsonins, C3b and iC3b for phagocytosis [24]. Activation of the complement terminal pathway results in releasing anaphylatoxins C5a, and formation of a membrane attack complex to lyse the target cells. Meanwhile, sC5b-9 as the final product of complement activation was significantly higher in CSF in CM group than that in the control group. Our results were similar with the report from Mook-Kanamori et.al [6], which demonstrated that CSF levels of C1q, MBL, FH and sC5b-9 were significantly elevated during pneumococcal and meningococcal meningitis. Thus our data suggest an important role of the complement system in host protection against CM as well. C1q, MBL and FB each separately initiates the classical, lectin and alternative pathway, and plays a key role in the innate immune system. C1q also take part in the clearance of apoptotic cells during inflammation resolution [25, 26]. Significantly elevated CSF levels of C1q, MBL and FB in CM patients may reflect all of the three pathway activations are involved in CM.

The differences of complement components between the CM and Ctrl groups were only detected in CSF samples, not in the plasma samples. This indicates that local inflammatory responses impact significantly on activity of the complement system in CSF. The brain as a well-developed organ of CNS is shielded by the blood–brain-barrier (BBB) with tight junction formations. BBB is a structural and functional barrier formed by the microvascular endothelium, astrocytes and pericytes. The complement system has taken part in various developmental and regenerative processes, and is integrated with multiple biological systems and pathways in many researches [27]. We think that the elevated CSF complement factors of patients with CM have leaked from the plasma because the CSF complement levels of C1q, MBL and FB were significantly correlated with the total concentrations of CSF protein. It is well known that a non-specific increase in permeability of the blood-CSF barrier during meningitis results in a rise in CSF protein concentrations including all the plasma proteins [28]. However, complement factors can be synthesized locally in the CNS [29]. Astrocyte and glial cell are the main sources of complement in CNS [30, 31]. Previous study using Reibergram method demonstrates that C3 and C4 were synthesized locally during bacterial meningitis. Further studies using Reibergram are needed to determine whether intrathecal synthesis of complement factors is, at least in part, responsible for the increased levels of CSF complement factors in CM.

However, complement has been described as a double-edged sword since activation of the complement system significantly contributes to the pathogenesis of various acute and chronic inflammatory diseases [32]. Adequate regulation of the complement system is a central component of the system’s ability to control unintended complement activation on host cells. The expression of regulator also can be detected in CSF, such as C4BP, FI, and FH. Their amounts are nearly the same in CSF and plasma. However, CSF FH concentrations were obviously higher in CM than that in the control group. FH regulates the AP both in the fluid phase and on cell surfaces. Expression of FH regulators indicates the balance also exits to control the degree of activation of complement. The role of the complement system activation in host defense and neuro-inflammation in CM is not fully understood. Elucidating which components of the complement system are important in both protective and inflammatory roles in CM to drive development of CM-specific immunotherapies.

It is well known that a Th1-driven cell-mediated response is necessary for the control of *C. neoformans* infections. Beenhouwer and his colleagues found that the relative inherent susceptibilities of the mouse strains to *C. neoformans* were as follows: *IL-12* ^*−/−*^ > *IL-6* ^*−/−*^ > C57BL/6 > > *IL-10* ^*−/−*^ [33]. In the present study, the CSF concentrations of IL-12 were significantly higher in CM group than that in Ctrl group. In mirror with other reports, our data indicated a protective role of IL-12 in CM. Exploring underlying mechanisms in regulation of IL-12 production and the cross talk with the complement system in CM would provide a new perspective for treatment of CM.

## Conclusion

In conclusion, our data revealed the presence of the complement components and the complement activation in CSF in non-HIV patients with CM, and suggested an important role of complement factors C1q, MBL, FB, FH and sC5b-9 in the pathogenesis of CM. Further studies are warranted to confirm the pathophysiological role of these complement factors in CM and to stimulate the development of new therapeutic strategies for CM by directing treatment to specific pathways within the complement cascade.

## Abbreviations

AP: Alternative pathway
BBB: Blood–brain-barrier
C1INH: C1 inhibitor
C4BP: C4 binding protein
CM: Cryptococcal meningitis
CNS: Central nervous system
CP: Classical pathway
CSF: Cerebrospinal fluid
Ctrl: Control patients
ELISA: Enzyme-linked immunosorbent assay
FB: Factor B
FH: Factor H
FI: Factor I
IL: Interleukin
LP: Lectin pathway
MBL: Mannose binding lectin
PVDF: Polyvinylidene difluoride
SDS-PAGE: Sodium dodecyl sulfate polyacrylamide gel electrophoresis
TBST: Tris-buffered saline
